# Prevalence of Dental Caries With Salivary Assessment in Six to Twelve Years Old School-Going Children in Shahpura Tehsil, Jaipur

**DOI:** 10.7759/cureus.27802

**Published:** 2022-08-08

**Authors:** Anita Choudhary, Manohar Bhat, Harinarayan Choudhary, Vivek Joshi, Satinder Singh Walia, Rajat K Soni

**Affiliations:** 1 Department of Dentistry, Jaipur Dental College, Jaipur, IND; 2 Department of Pediatric and Preventive Dentistry, Jaipur Dental College, Maharaj Vinayak Global University, Jaipur, IND; 3 Department of Orthodontics and Dentofacial Orthopedics, National Institute of Medical Science (NIMS) Medical University, Jaipur, IND; 4 Department of Dentistry, Creative Head Consultants, Jaipur, IND; 5 Department of Public Health Dentistry, Sri Guru Ram Das Institute of Dental Sciences and Research, Jaipur, IND; 6 Department of Orthodontics and Dentofacial Orthopedics, Jaipur Dental College, Jaipur, IND

**Keywords:** salivary buffering capacity, dmft index, salivary ph, salivary flow rate, dental caries

## Abstract

Introduction

Dental caries is a chronic, infectious, and irreversible disease of the calcified tissues of teeth, which demineralises the inorganic portion and destroys the organic substance of the tooth, which often leads to cavitation. Epidemiological studies measuring the prevalence and severity of dental caries have used modified versions of Klein and colleagues’ decayed, missing, and filled (DMF) or Gruebbel’s decayed, extraction indicated, and filled (def) indexes; however, these indexes only capture cavitated lesions. Saliva plays a vital role in caries prevention; significant reduction or deterioration of salivary function can aggravate the development of dental caries. Saliva affects the incidence of dental caries in four ways: as a mechanical cleansing agent that results in less accumulation of plaque, by reducing enamel solubility using calcium, phosphate, and fluoride, by buffering and neutralising the acids produced by cariogenic organisms, or by introducing directly through diet and by antibacterial activity. The study aims to assess the prevalence of dental caries and salivary parameters such as salivary pH, flow, and buffering capacity in six to 12 years old children of a rural tehsil of Jaipur.

Materials and methods

The study was done on a population consisting of 400 school-going children in the age group of six to 12 years. Oral examination was undertaken by a single examiner, who is the study's principal investigator, to avoid inter-examiner variability. Testing of resting saliva was done for evaluation of visual inspection of the level of hydration, saliva consistency, pH measurement, saliva quantity, and buffering capacity.

Statistical analysis

The data were analysed using the chi-square test, t-test, and statistical software SPSS version 17.00 (SPSS Inc., Chicago, IL). The chi-square test was used to compare and analyse qualitative data, whereas the unpaired t-test was used to analyse and compare quantitative data. Quantitative data were summarised as mean and standard deviation. A p-value of 0.001 or less was considered for standard significance.

Results

There was no significant difference in resting salivary flow rate between children with decayed, missing, and filled teeth (DMFT) scores less than 5 and DMFT scores of 5. The mean buffering capacity of stimulated saliva was found to be significantly more among children with DMFT scores less than 5 than children with DMFT scores of 5 or more. The mean pH of resting saliva was found to be significantly higher among children with DMFT scores less than 5 than children with DMFT scores of 5 or more.

Conclusion

The prevalence of caries based on age was maximum in mixed dentition and minimum in primary dentition. In contrast, the difference in severity based on age was maximum in permanent dentition. The prevalence of caries was higher in children whose parents were aware of dental health; the difference was more significant in children with primary and mixed dentition. This study showed that salivary parameters such as salivary flow rate, salivary pH, and salivary buffering capacity among school-going children correlated with the prevalence of caries.

## Introduction

Dental caries is a chronic, infectious, and irreversible disease of the calcified tissues of teeth, which demineralises the inorganic portion and destroys the organic substance of the tooth, which often leads to cavitation [1]. The word "caries" is derived from a Latin word meaning "rot" or decay. It is a complex and dynamic process where many factors influence and initiate disease progression [2].

Epidemiological studies measuring the prevalence and severity of dental caries have used modified versions of Klein and colleagues’ decayed, missing, and filled (DMF) or Gruebbel’s decayed, extraction indicated, and filled (def) indexes; however, these indexes only capture cavitated lesions [3]. The pervasiveness of dental caries in India is 50-60%. An interplay of three principal factors is responsible for this multi-factorial disease host: teeth and saliva, microorganisms in the form of dental plaque, and substrate (diet).

Thus, caries requires a susceptible host, cariogenic oral flora, and a suitable substrate, which must be present for a sufficient length of time [4]. Saliva is a biologic fluid in the oral cavity, comprised of a mixture of secretory products from the major and minor salivary glands [5]. Saliva plays an essential role in caries prevention; significant reduction or deterioration of salivary function can contribute to the progression of dental caries. Saliva affects the incidence of dental caries in four ways: as a mechanical cleansing agent that results in less accumulation of plaque, by reducing enamel solubility by utilising calcium, phosphate, and fluoride, by buffering and neutralising the acids produced by cariogenic organisms, or by introducing directly through diet and by antibacterial activity [6]. The main aim of this research is to assess the prevalence of dental caries and salivary parameters such as salivary pH, flow, and buffering capacity in six to 12 years old children of a rural tehsil of Jaipur.

## Materials and methods

The study was done in the Department of Pedodontics and Preventive Dentistry, Jaipur Dental College, Jaipur, on a population consisting of 400 school-going children in the age group of six to 12 years from schools in Shahpura (around Jaipur city), which includes both male and female children. Participants were selected by random sampling method. Before beginning the fieldwork, a project program was written and presented to the ethical committee of the college, which accepted the investigation with the number JDC/IDSH/963/17.

Data collection

Information and lists of governments and private schools in Shahpura were obtained from the "block education officer". Eight schools were selected; two schools in one direction (two north, two south, two west, and two east). Inclusion criteria were children aged six to 12 years per age specification record given by involved school authorities, while exclusion criteria comprised children below six and above 12 years of age, children who were severely ill, children who had taken antibiotics in the last months, and children using orthodontic appliances.

Method of examination

To avoid inter-examiner variability, a single examiner, the study's principal investigator, undertook an oral examination. The recording was done by a trained person who assisted the examiner throughout the study. Chemical sterilisation (glutaraldehyde 2.4%) was used to sterilise the instruments. Buffer kit (GC India, Telangana, India) was used to report, identify, measure, and assess caries risk based on saliva.

Visual inspection of the level of hydration

**Figure 1:**
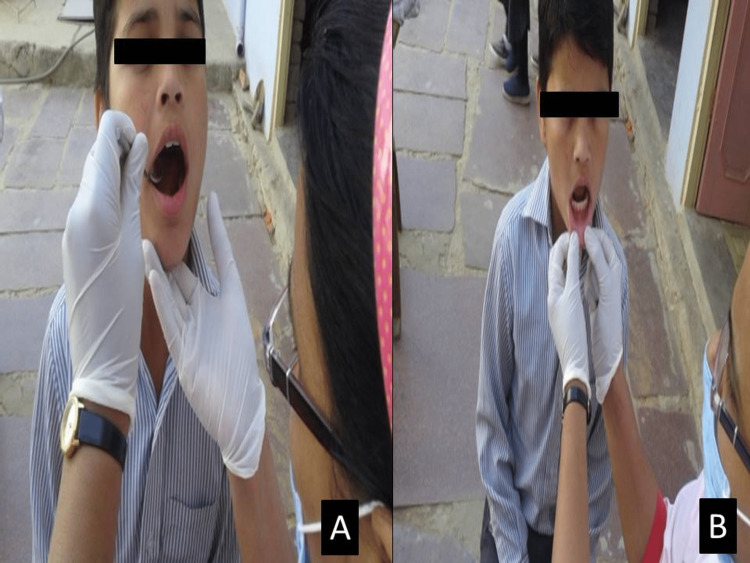
Clinical examination of the salivary flow (drop on lower lip) (A) Oral examination. (B) Saliva droplets on the lower lip.

Visual assessment of the lower lip labial gland secretion was done by everting the lower lip, gently blotting the labial mucosa with a small piece of gauze, and observing the mucosa under natural light. Saliva droplets will form at the orifices of minor glands. Assess the time for visible production of saliva as follows: if greater than 60 seconds, then resting flow is low, and if less than 60 seconds, then resting flow is normal (Figure 1).

Saliva consistency

On the visual assessment of the resting salivary consistency in the oral cavity, if the saliva appears to be sticky and frothy, it indicates increased viscosity, frothy and bubbly saliva indicates increased viscosity, and watery clear saliva implicates normal viscosity.

pH measurement

**Figure 2:**
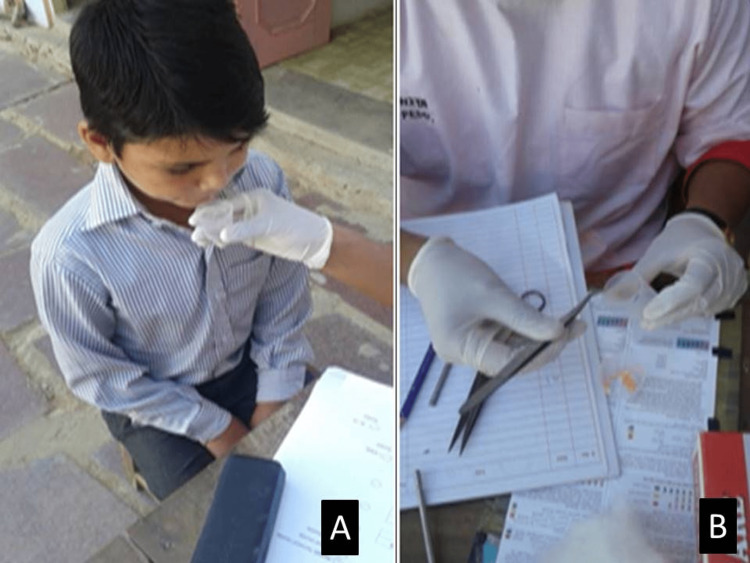
Collection of saliva in a cup and dipping the pH strip in saliva (A) Collection of saliva. (B) pH strips.

Pooled saliva was collected in the collection cup, pH test strips were placed in the resting saliva for 10 seconds, and then the strip's colour was checked. Finally, it was compared with the testing chart available in the package (Figure 2).

Saliva quantity

The children were instructed to chew on a piece of wax to stimulate salivary flow. After 30 seconds, the child was asked to expectorate into the spittoon. Chewing was continued for a further five minutes, collecting all the saliva into the collection cup at regular intervals. The quantity of saliva was measured by checking the millilitre markings on the side of the cup. If the quantity of saliva at five minutes is more diminutive than 3.5 mL, then the production is very low; if it is between 3.5 and 5.0 mL, then it is low, and if it is more than 5.0 mL, then the production is average. The regular stimulated saliva flow rate may range between 1 and 1.6 mL/min.

Buffering capacity

**Figure 3:**
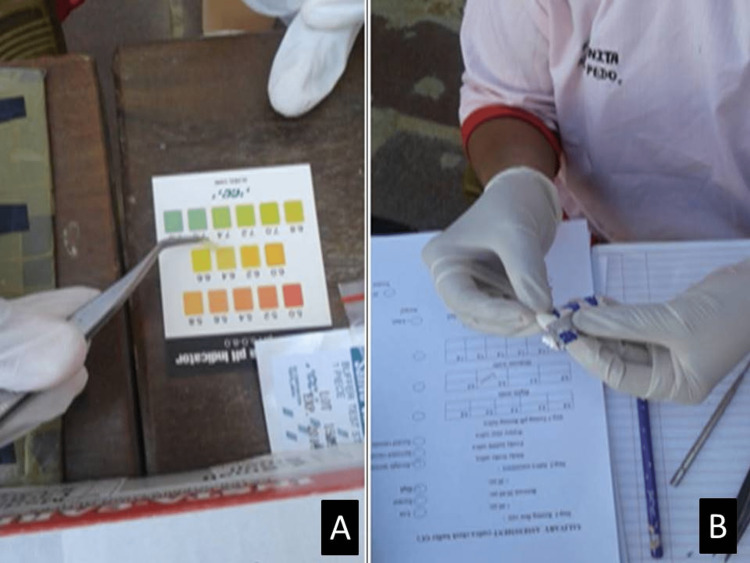
Matching the strip colour with foil package for buffer test using pH pad (A) Buffer test pH pad. (B) Matching the strip colour with foil package.

The buffering capacity is measured as follows: take a buffer test strip from the foil package and place it on the absorbent tissue with the test side up. Draw sufficient saliva from the collection cup with the help of a pipette and dispense one drop on each of the three test pads. Turn the strip 90 degrees to soak up excess saliva from swelling on the test pad, possibly affecting the test result's accuracy. The test strips will begin to change colour immediately. After two minutes, the final result can be calculated by adding the points according to the final colour of each pad (Figure 3).

Clinical examination/oral examination

Examination of subjects was performed on an upright chair in adequate natural daylight. Dental caries is measured by the decayed, missing, and filled teeth (DMFT) index (Henry, Palmer, and Knutson 1938). Carious lesions were detected at the cavitation level with the visual and tactile method. In the next part of our study, a questionnaire was constructed and was given to each subject. The questionnaire was easy to understand and had various domains to gain knowledge about the subject's feeding practice, oral hygiene, dietary pattern, and attitude towards dental treatment. The parents of children included in the study collected the questionnaire the next day after completion. The questionnaire was provided in Hindi and English language for ease of comprehension.

Statistical analysis

The data were analysed using the chi-square test, t-test, and statistical software SPSS version 17.00 (SPSS Inc., Chicago, IL). The chi-square test was used to analyse and compare qualitative data, whereas the unpaired t-test was used to analyse and compare quantitative data. Quantitative data were evaluated as mean and standard deviation. A p-value of 0.001 or less was considered for standard significance. The correlation between two variables was calculated by using the Pearson correlation coefficient.

## Results

This study evaluated the prevalence of dental caries with salivary assessment in six to 12 years old school-going children in and around Shahpura Tehsil, Jaipur. The examination included 400 school-going children aged six to 12 years. The prevalence of dental caries in general and different directional areas is shown in Table 1.

**Table 1:** DMFT score DMFT: decayed, missing, and filled teeth.

	DMFT score			
	Minimum	Maximum	Mean	Std. deviation
Overall	0	12	2.34	2.41
North	0	9	2.25	2.43
South	0	12	2.02	2.46
East	0	11	2.76	2.26
West	0	12	2.34	2.48

The chi-square test compared the resting salivary flow rate between children with DMFT scores less than 5 and DMFT scores of 5 or more. There was no significant difference in resting salivary flow rate between children with DMFT scores less than 5 and DMFT scores of 5 (Table 2).

**Table 2:** Salivary assessment of the overall resting flow rate Chi-square test. Chi-square value = 2.446 and p-value = 0.294^#.^ #: non-significant difference; DMFT: decayed, missing, and filled teeth.

Resting flow rate	DMFT less than 5	DMFT 5 or more	Total
High	108	18	126
	32.8%	25.4%	31.5%
Low	53	16	69
	16.1%	22.5%	17.3%
Normal	168	37	205
	51.1%	52.1%	51.3%
Total	329	71	400
	100.0%	100.0%	100.0%

The mean buffering capacity of stimulated saliva was compared between children with DMFT scores less than 5 and DMFT scores of 5 or more using the unpaired t-test. The mean buffering capacity of stimulated saliva was found to be significantly more among children with DMFT scores less than 5 than children with DMFT scores of 5 or more (Table 3).

**Table 3:** Salivary assessment of the overall buffering capacity of stimulated saliva Unpaired t-test. *: significant difference; DMFT: decayed, missing, and filled teeth.

DMFT	Buffering capacity of stimulated saliva				
	Mean	Std. deviation	Mean difference	t-test value	P-value
DMFT less than 5	6.44	0.68	1.64	18.55	<0.001*
DMFT 5 or more	4.79	0.67			

The Pearson correlation test assessed the correlation between DMFT score and buffering capacity of stimulated saliva. There was a negative correlation between DMFT score and buffering capacity of stimulated saliva. As the buffering capacity of saliva decreased, DMFT scores increased (Table 4).

**Table 4:** Buffering capacity of stimulated saliva correlated to DMFT score Pearson’s correlation test. *: correlation is significant at the 0.01 level; DMFT: decayed, missing, and filled teeth.

		DMFT score
Buffering capacity of stimulated saliva	Pearson correlation	-0.894
	P-value	<0.001*
	N	400

The mean pH of resting saliva was compared between children with DMFT scores less than 5 and DMFT scores of 5 or more using the unpaired t-test. The mean pH of resting saliva was found to be significantly higher among children with DMFT scores less than 5 than children with DMFT scores of 5 or more (Table 5).

**Table 5:** Salivary assessment of the overall pH of resting saliva Unpaired t-test. *: significant difference; DMFT: decayed, missing, and filled teeth.

	pH resting saliva				
DMFT	Mean	Std. deviation	Mean difference	t-test value	P-value
DMFT less than 5	7.09	0.44	0.41	7.450	<0.001*
DMFT 5 or more	6.68	0.34			

The Pearson correlation test assessed the correlation between DMFT score and pH of resting saliva. There was a significant negative correlation between DMFT score and pH of resting saliva. As the pH of resting saliva decreased, the DMFT score increased (Table 6).

**Table 6:** pH of resting saliva correlated to DMFT score Pearson’s correlation test. *: correlation is significant at the 0.01 level; DMFT: decayed, missing, and filled teeth.

		DMFT score
pH resting saliva	Pearson correlation	-0.354
	P-value	<0.001*
	N	400

## Discussion

This study evaluated the prevalence of dental caries in a sample of 400 school-going children aged six to 12 years in and around Shahpura Tehsil, Jaipur, Rajasthan. Random sampling methods were followed. The overall DMFT score and deviation were 2.34 (2.41), while zonal DMFT score and deviation were as follows: north: 2.25 (2.43); south: 2.02 (2.46); east: 2.76 (2.26); and west: 2.34 (2.48). Previous studies stated that the highest caries prevalence was seen in children aged eight to nine years, followed by 10-12 and six to seven years [7].

Pandit et al. studied the prevalence of dental caries in a mixed dentition period among six to 12 years old and found that the mean DMFT score for six to seven years was 1.70, while 1.17 for the age group of eight to nine years and 0.77 for the age group of 10-12 years [8].

Epidemiologic studies measuring the prevalence and severity of dental caries have used modified versions of Klein and colleagues’ decayed, missing, and filled (DMF) or Gruebbel’s decayed, extraction indicated, and filled (def) indexes [9].

Mohamedhussein et al. assessed the caries risk in children aged five to eight years using salivary parameters such as salivary flow, pH, buffer capacity, and bacterial count. The mean DMFT was 10.67 [5].

Salivary flow rate

In this study, there was no significant difference in resting salivary flow rate between children with DMFT scores less than 5 and DMFT scores of 5 or more. In addition, no correlation was found between salivary flow rate and caries index. These results correlate with the study done by Garan et al. in which the DMFT score was found to be lower in children with black stains compared with those without stains, and there was no relationship between salivary parameter and caries indices in children [10].

Salivary pH

Prabhakar et al., Pruthi et al., Mohammadi et al., Gopinath et al., and Mohamedhussein et al. found that the correlation between pH and caries index was not statistically significant. However, in this study, there was a significant difference in the mean salivary pH among study groups (p < 0.001) [5,11-14]. In addition, the mean pH of resting saliva was found to be significantly higher among children with DMFT scores less than 5 than children with DMFT scores of 5 or more.

Salivary buffering capacity

Khan et al. found a significant positive correlation between buffering capacity and caries index, which is in accordance with this study; there was a significant difference in mean salivary buffering capacity among the study groups (p < 0.001) [15]. In addition, the mean buffering capacity of stimulated saliva was found to be significantly more among children with DMFT scores less than 5 than children with DMFT scores of 5 or more.

The present study's main limitation was that all data were obtained by interviewing children through questionnaires due to the lack of parental collaboration. Therefore, the information obtained regarding sugar intake and brushing frequency may not be entirely reliable. Therefore, it would be necessary to carry out a study with a more significant sample number and give out the questionnaires to parents.

## Conclusions

The overall DMFT score in six to 12 years old school-going children was more than 2 and less than 3. The prevalence of caries based on age was maximum in mixed dentition and minimum in primary dentition. In contrast, the difference in severity based on age was maximum in permanent dentition. According to the results obtained from the questionnaire, the prevalence of caries was more significant in children with primary and mixed dentition. There was no significant difference in resting salivary flow rate between children with DMFT scores less than 5 and DMFT scores of 5 or more (p = 0.294). The mean pH of resting saliva was found to be significantly higher among children with DMFT score less than 5 compared to children with DMFT score of 5 or more; resting saliva pH decreased and DMFT score increased. This study showed that salivary parameters such as salivary pH and salivary buffering capacity among school-going children correlated with the prevalence of caries.
